# Development of an Automated Imaging Pipeline for the Analysis of the Zebrafish Larval Kidney

**DOI:** 10.1371/journal.pone.0082137

**Published:** 2013-12-04

**Authors:** Jens H. Westhoff, Stefan Giselbrecht, Miriam Schmidts, Sebastian Schindler, Philip L. Beales, Burkhard Tönshoff, Urban Liebel, Jochen Gehrig

**Affiliations:** 1 Department of Pediatrics I, University Children’s Hospital, University of Heidelberg, Heidelberg, Germany;; 2 Institute for Biological Interfaces 2, Karlsruhe Institute of Technology, Eggenstein-Leopoldshafen, Germany; 3 Molecular Medicine Unit, Birth Defects Research Centre, University College London (UCL), Institute of Child Health, London, United Kingdom; 4 Institute for Applied Computer Science, Karlsruhe Institute of Technology, Eggenstein-Leopoldshafen, Germany; 5 Institute of Toxicology and Genetics, Karlsruhe Institute of Technology, Eggenstein-Leopoldshafen, Germany; 6 Accelerator Laboratory, Innovation Department, Karlsruhe Institute of Technology, Eggenstein-Leopoldshafen, Germany;; Institute of Cellular and Organismic Biology, Taiwan

## Abstract

The analysis of kidney malformation caused by environmental influences during nephrogenesis or by hereditary nephropathies requires animal models allowing the *in vivo* observation of developmental processes. The zebrafish has emerged as a useful model system for the analysis of vertebrate organ development and function, and it is suitable for the identification of organotoxic or disease-modulating compounds on a larger scale. However, to fully exploit its potential in high content screening applications, dedicated protocols are required allowing the consistent visualization of inner organs such as the embryonic kidney. To this end, we developed a high content screening compatible pipeline for the automated imaging of standardized views of the developing pronephros in zebrafish larvae. Using a custom designed tool, cavities were generated in agarose coated microtiter plates allowing for accurate positioning and orientation of zebrafish larvae. This enabled the subsequent automated acquisition of stable and consistent dorsal views of pronephric kidneys. The established pipeline was applied in a pilot screen for the analysis of the impact of potentially nephrotoxic drugs on zebrafish pronephros development in the *Tg*(*wt1b:EGFP*) transgenic line in which the developing pronephros is highlighted by GFP expression. The consistent image data that was acquired allowed for quantification of gross morphological pronephric phenotypes, revealing concentration dependent effects of several compounds on nephrogenesis. In addition, applicability of the imaging pipeline was further confirmed in a morpholino based model for cilia-associated human genetic disorders associated with different intraflagellar transport genes. The developed tools and pipeline can be used to study various aspects in zebrafish kidney research, and can be readily adapted for the analysis of other organ systems.

## Introduction

Adverse environmental conditions and genetic influences can have a major impact on organogenesis. According to statistical surveys, 22-50% of all pregnant women take drugs within the first trimenon of gestation, often whilst they are unaware of their pregnancy [1]. However, nephrotoxic medication taken by pregnant women or administered to preterm newborns with ongoing nephrogenesis can interfere with nephron generation and thus can cause short- and potentially long-term kidney impairment [2]. Besides environmental factors, hereditary nephropathies due to mutations in genes encoding ciliary proteins are the most common cause of end-stage renal disease in children due to polycystic, cystic-dysplastic and/or nephronophthisis like renal phenotypes [3]. 

Despite rodent studies and observational reports in humans, detailed data on potential harmful side effects on nephrogenesis is still missing for many drugs and chemical compounds [4,5]. Additionally, there is a lack of large-scale chemical screens for modifiers of hereditary nephropathies. This is mainly due to the very laborious and time-consuming testing of substance specific effects on normal and abnormal organogenesis using standard experimental setups, thus hampering investigations on a larger scale. Additionally, *in vivo* imaging of kidney development in a spatiotemporal context is not feasible in rodents, necessitating animal models that are experimentally more accessible. Furthermore, dedicated imaging techniques are required which enable the *in vivo* visualization of developing organs and tissues on a larger scale.

The development of high content screening (HCS) technologies has had a major impact on biomedical and pharmaceutical research, as these platforms are suitable for a wide range of large-scale investigations using *in vitro* and *in vivo* model systems [6]. Due to its small size and various other experimental advantages, the zebrafish can be readily employed in large scale *in vivo* assays. Moreover, the high degree of anatomical and physiological homology to higher vertebrates renders it a relevant model for biomedical research [7]. Thus, the zebrafish has emerged as the main vertebrate model system for whole organism screening experiments. Consequently, the zebrafish has been successfully used in chemical, toxicological, behavioral and genetic screening experiments [4,7 8 9 10–11]. Moreover, its transparency in combination with the wealth of mutant and transgenic zebrafish strains available facilitates the large scale analysis of tissue-specific phenotypes. This includes, for example, the search for anti-inflammatory, anti-angiogenic or neuroactive compounds [12 13–14].

Despite its widespread usage in large scale experiments, it remains challenging to fully exploit the advantages of this model system in HCS experiments. Zebrafish embryos are largely incompatible with standard HCS protocols developed for other model systems, one of the reasons being that their relatively large size and complex three-dimensional shape often lead to random positioning and orientation of embryos within wells of microtiter plates. This especially hampers reproducibility and detailed visualization of cell or tissue morphology, as well as developmental processes. Whilst several studies and recent technological advances have tried to address these challenges, they often require sophisticated technical setups [15] or are incompatible with chemical screening [16]. Thus, there remains a demand of easy-to-use screening protocols and dedicated tools which can facilitate performing complex zebrafish HCS assays.

In the zebrafish larva, the functional pronephros comprises only 2 nephrons with fused glomeruli located ventrally to the dorsal aorta. Interestingly, despite tremendous differences in nephron number, the composition of a single nephron shows great homology at the cellular and molecular level between human and zebrafish [17,18]. Additionally, kidney development and function largely depend on the same orthologous genes for all vertebrate kidneys. Therefore, studying formation and function of the zebrafish pronephros can aid in the understanding of the role of genes mutated in kidney disease, or the impact of compounds on renal development and function in humans [17]. Thus, the combination of this *in vivo* model system with automated imaging technologies could serve as a tool for the large scale analysis of kidney phenotypes. However, to our current knowledge, a screening platform compatible with *in vivo* imaging of zebrafish larval kidneys has not been described yet.

Here, we delineate the development of an automated HCS compatible imaging pipeline designed for live imaging of zebrafish kidneys in chemical screening scenarios. Using a custom designed orientation tool, embryos could be accurately positioned in wells of microtiter plates allowing consistent imaging of dorsal views of the pronephros. Subsequent automated imaging was performed on a standard widefield screening microscope and a data handling and visualization pipeline was developed. A pilotscreen for morphological kidney abnormalities was performed using a subset of potentially nephrotoxic drugs applied to larvae of the *Tg*(*wt1b:EGFP*) transgenic line in which the developing pronephros is highlighted by GFP expression [19]. The obtained *in vivo* data was cross-validated by histological analysis. In addition, we demonstrate that the established microscopy platform can also be utilized for genetic disease models.

## Materials and Methods

### Ethics statement

All zebrafish husbandry and experimental procedures were performed in accordance with the German animal protection standards and were approved by the Government of Baden-Württemberg, Regierungspräsidium Karlsruhe, Germany (Aktenzeichen 35-9185.64).

### Fish keeping and embryo handling

Adult zebrafish of the *Tg*(*wt1b:EGFP*) transgenic line [19] were maintained according to reference [20]. Eggs were collected from pairwise and batch crossings. The developmental stage of embryos was determined as previously described [21]. Embryos were raised in fish water at 28°C. At 24 hpf embryos were enzymatically dechorionated using 10 mg/ml Pronase. Embryos were transferred to a beaker, washed twice with 400 ml of fish water and transferred into clean petri dishes [22]. Prior to transferring into agarose coated microtiter plates, 48 or 72 hpf old larvae were anesthetized using 0.03% tricaine.

### Drug treatment of embryos

A subset of certain drug classes was chosen for which an adverse effect on the developing kidney had been described in animal and/or human studies [2]. To evaluate concentration-dependent toxicity, 5 different concentrations of each drug (2.5 mM, 5 mM, 10 mM, 20 mM, 40 mM) were tested. 24 hpf dechorionated embryos were transferred to 6-well-plates and treated with the following drugs dissolved in E3 solution with 0.003% 1-pheny-2-thiourea (PTU, Alfa Aesar, Karlsruhe, Germany): penicillin G potassium salt (AppliChem, Darmstadt, Germany), ampicillin sodium salt (AppliChem, Darmstadt, Germany), gentamicin sulfate (Sigma-Aldrich, St. Louis, USA), kanamycin sulfate (AppliChem, Darmstadt, Germany), captopril (CalBiochem, Darmstadt, Germany), losartan potassium salt (Molekula, Gillingham, Dorset, United Kingdom), acetaminophen (Caesar und Loretz, Hilden, Germany), indomethacin sodium salt (AppliChem, Darmstadt, Germany). Treatment period was 24 hours. For indomethacin, lower concentrations (0.01 mM, 0.025 mM, 0.05 mM, 0.075 mM and 0.1 mM) had to be applied due to 100% lethality rates at higher concentrations. 2-4 repeats of each experiment were performed. Total number of embryos that underwent drug treatment is shown in Table **S1**. The pH was adjusted for each experiment. Following drug treatment, the number of dead larvae was assessed and the living larvae were transferred to 0.003% PTU containing E3 solution. For imaging studies, 0.03% tricaine was added to the medium.

### Morpholino injections

Antisense splice blocking morpholino oligonucleotides (Gene Tools, LLC, Philomath, USA) were designed against the exon1-intron1 and exon2 –intron2 boundary of zebrafish *ift172* gene, and ift80 morpholino has been previously published [23]. Morpholinos were injected into 1-cell stage embryos at 500 nM concentration. Both ift172 morpholinos resulted in similar phenotypes comparable to the phenotype previously published [24 25–26]. A standard control oligonucleotide was used as control. Morpholino sequences: ift80 splice blocking MO: 5’-AGGTGTATGTGGAACCTGTGATAAG-3’, ift172 Exon2 splice donor blocking MO: 5’-ACGAGATGAGAGCTTACTTTTGGGT-3’, ift172 Exon1 splice blocking MO: 5’-ACGCTGTCAATATTTTACCTGAGGC-3’, Standard control (ctr) oligonucleotide: 5’-CCTCTTACCTCAGTTACAATTTATA-3’.

### Generation of a 96 well template tool

The tool is made from brass and consists of a base plate with 96 perpendicular pins arranged in a certain way to match the positions of the wells of a microtiter plate. The tool was produced by CNC milling (Datron, M7). In a first step, the base plate with the dimensions of 140 mm x 100 mm x 21 mm was milled by a four flute end mill (20 mm). Secondly, the array of 96 pins with 11 mm height and rectangular footprint (5.9 mm x 1 mm) was created by partly thinning the base plate down to 9 mm between the pins (Four Flute End Mill, 6 mm and 3 mm, respectively). Finally, the ends of the 96 pins were tapered to a conical tip with 60° by utilizing a standard engraving tool. 

### Preparation of agarose molds in microtiter plates

70 µl of 1% agarose in fish water was added to each well of a 96 well microtiter plate (Cat.-No. 655180, Greiner, Frickenhausen, Germany) using a multi-channel pipette. The agarose coated well plates were pre-cooled at room temperature for one minute. To generate grooves the brass tool was inserted, while adjusting the penetration depth of pins using spacers. After solidification the tool was carefully removed and plates were optionally stored in plastic bags at 4°C. Embryos were transferred in 100 µl fish water containing PTU and tricaine (see above) and manually arrayed and oriented under a stereomicroscope. To aid in positioning of regions of interest within the grid-based and fixed field views of the Scan^R system, embryos were positioned in such a way that all yolk sacs were approximately at the same position within cavities using features of the well plate as guidelines. 

### Image acquisition

Embryos were imaged on a standard Scan^R high-content screening microscope (Olympus, Hamburg, Germany) [27] as previously described [16,22]. Data was acquired using 33 z-slices (Δz = 15μm) per embryo and channel using a 4x (N.A. = 0.13) objective. Integration times were fixed (80 ms for GFP). Imaging times were approximately 1 hour for a full 96 well plate. For each experimental plate the A1 positions of the imaging grid was re-centered to compensate for minor differences in positioning of embryos. 

### Data handling, deconvolution and visualization

Data handling, generation of multilayer tiffs and generation of maximum projections were carried out using custom written Perl scripts and Fiji [28] macros available on request. Fluorescence z-stacks were deconvolved with Huygens Professional deconvolution software (SVI, Hilversum, The Netherlands) using a theoretical point spread function based on microscope parameters. Batch deconvolution was carried out on a workstation with 24 CPU cores and 64 Gigabyte of memory. Cropping of images and generation of overview images was carried out using a Fiji macro (**Macro S1**). In brief, maximum projection images were duplicated, automatically thresholded and the resulting binary images were eroded. The position of kidneys was detected by measuring the center of mass. The corresponding coordinates were restored on original images, a bounding box was defined and images were cropped accordingly. Cropped images were loaded into a stack and overview images were generated using the ‘Make Montage’ function of Fiji. 

### Analysis of morphological and pronephric phenotypes

Overall morphology was scored on a Leica MZ10 F stereomicroscope (Leica Microsystems, Wetzlar, Germany). Lethality and pericardial/yolk sac edema were rated for each experiment. Blinded analysis of tubular and glomerular phenotypes was performed by SS and JHW on maximum projections of deconvolved z-stacks. Using ImageJ, 2 parameters were manually measured for tubular structures: i) maximum distance between the tubules and ii) angle between neck segment and proximal convoluted tubule [18] being visible in *Tg*(*wt1b:EGFP*) zebrafish. For description of glomerular changes, the distance between the glomeruli was determined and glomeruli were classified as either normal or showing glomerular malformation judged by the glomerular area and structure. Heatmaps were generated using matrix2png [29].

### Histological analysis

Larvae were fixed in 4% paraformaldehyde overnight at 4°C. Samples were dehydrated through an ethanol series and processed for embedding in Paraffin (Surgipath® Paraplast®, Leica Biosystems, Wetzlar, Germany). 3 μm sections were cut using a Leica RM 2165 microtome (Leica Microsystems, Nussloch, Germany). Sections were deparaffinized in xylene and rehydrated through graded washes of ethanol in water before staining with hematoxylin and eosin. The stained sections were imaged with a Leica DMI4000 B microscope equipped with a Leica DFC320 digital camera.

### Statistical analysis

Data were evaluated using IBM® SPSS® Statistics Version 21. For lethality and edema rates and glomerular fusion and malformation, statistical analysis was performed by Chi-square test. Datasets of low sample sizes were additionally tested using Fisher’s exact test. For tubular angle and distance, means among treatment groups were compared using ANOVA with Bonferroni correction for multiple comparisons as a post-hoc test. Significance was defined as p<0.05. 

## Results and Discussion

### Standard positioning of embryos for chemical screening

The visualization of bilateral symmetric organs of zebrafish embryos usually demands dorsal or ventral views. However, consistent large scale imaging of zebrafish embryos remains challenging in automated screening experiments, as stable and reproducible positioning is complicated by the size and complex three-dimensional shape of embryos. 

**Figure 1:**
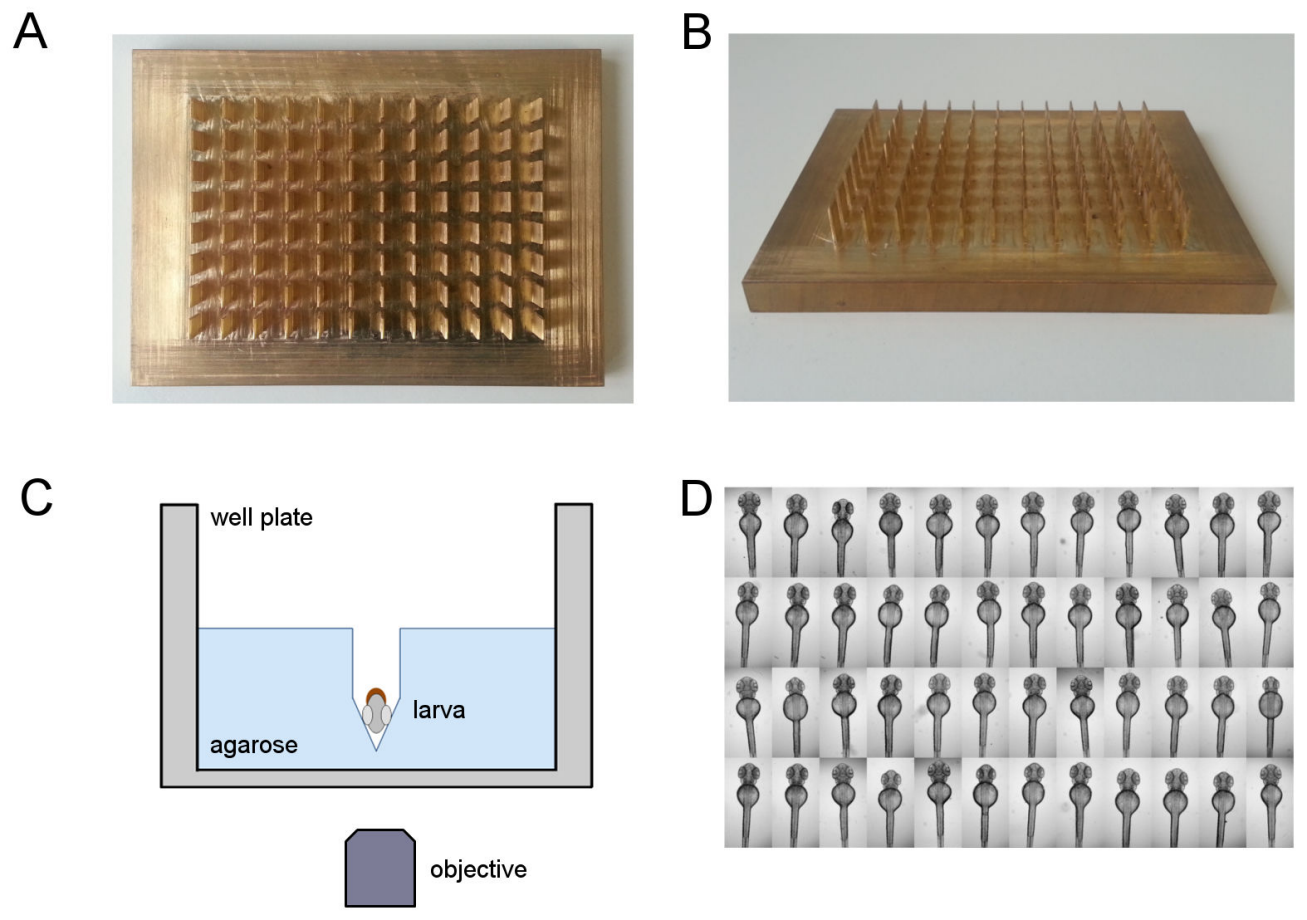
Standardized orientation of zebrafish embryos. (A,B) Photographs of the brass tool for the simultaneous generation of agarose grooves within 96 well microtiter plates: (A) top view and (B) tilted view. For dimensions of the plate see Materials and Methods section. (C) Schematic depiction of a single well with a ventrally oriented embryo within an agarose cavity. Drawing is not to scale. (D) Illustrative example of aligned and oriented embryos. Shown are dorsal views of 48 hpf embryos acquired using a 2.5x objective on an inverted wide field screening microscope.

To enable a simplified handling and precise positioning of zebrafish larvae and to achieve consistent visualization of tissues in high content screening scenarios, we have developed a tool to create agarose molds in a standard microtiter plate. The tool allows the preparation of agarose coated 96 well plates in a single replication step. The tool consists of a base plate with 96 rectangular pins, whose positions exactly match the centers of wells of standard and commercially available 96 well microtiter plates (Figure **1A, B**). The pins end with a keel shaped geometry, which was previously shown to be suitable for accurate ventral positioning of zebrafish larvae [16]. The tool was fabricated out of a solid block of brass using CNC milling (Figure **1A, B**), giving rise to a precise work piece with identical xyz-dimensions of each pin allowing the generation of deep keel-shaped cavities in wells of a 96-well plate filled with agarose (Figure **1C**). Such prepared plates can be readily used to manually position specimen enabling the subsequent automated acquisition of dorsal views using inverted screening microscope systems (Figure **1D**).

We recently demonstrated an alternative protocol for automated dorsal imaging of oriented larvae using a silicone tool to generate an array of 96 agarose molds [16]. However, while plates generated with this tool can be employed for the consistent automated imaging of tissues, the design prevents utilization in chemical or drug screening applications. The novel device allows generation of molds in each well independently, thus avoiding cross-contamination, which was a major limitation of the previous design. Furthermore, the restriction to single wells and the depths of the cavities drastically minimizes movement of surrounding medium leading to a massively improved stability of orientation of embryos in comparison to the silicone template. Thus, plates with oriented embryos and larvae could be used in combination with automated plate handling and stacking systems. 

### Pipeline for automated pronephros imaging

**Figure 2:**
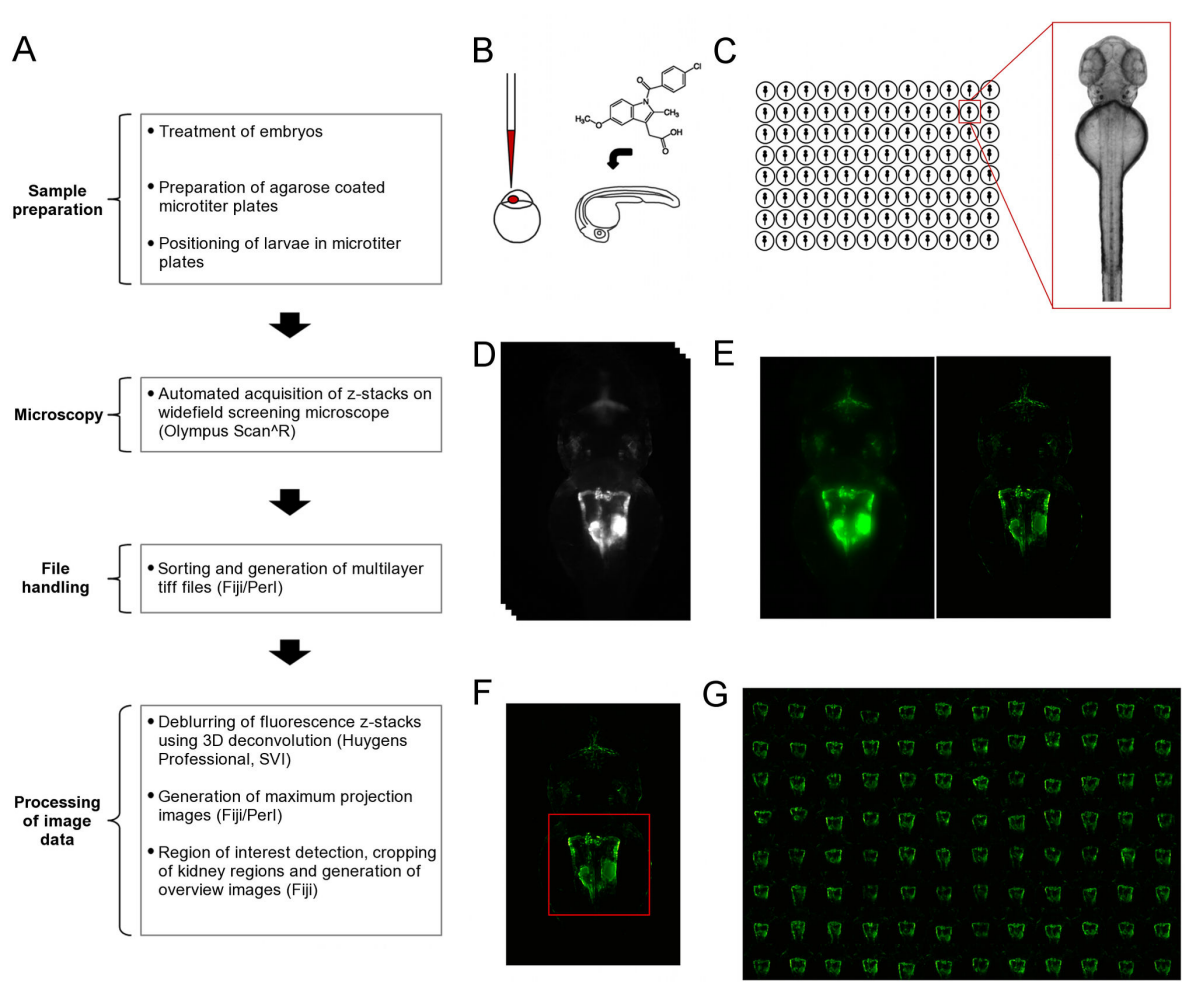
Overview of workflow for the automated imaging of the developing zebrafish pronephros. (A) Overview of the workflow for screening larval kidneys. The flowchart illustrates the different steps carried out to obtain overview images of kidneys. (B) Initial compound treatment or microinjection of embryos prior to sample preparation and imaging. (C) Schematic illustrating the transfer of embryos into agarose coated microtiter plates, and alignment and orientation of embryos. (D-G) Acquisition and processing of image Data. D to F show data of the same embryo. (D) Automated acquisition of z-stacks (33 z-slices, dZ=15µm) on an inverted widefield screening microscope. (E) Deblurring of images using deconvolution. Shown are maximum projections of z-stacks of raw data (left panel) and deconvolved data (right panel). (F) Automated detection and cropping of the kidney region. The red square indicates the position and dimensions of the cropped region. (G) Automated generation of overview images for quick assessment of overall morphological changes. Indomethacin skeletal formula in (A) taken from (http://en.wikipedia.org/wiki/File:Indometacin_skeletal.svg).

To visualize and score renal phenotypes, we developed a protocol for automated imaging of dorsal views of zebrafish larval kidneys (Figure **2A**). Prior to imaging, compound treated or microinjected embryos of the *Tg*(*wt1b:EGFP*) stable transgenic line were raised to the desired developmental stage (48 or 72 hpf) and then transferred into microtiter plates containing agarose cavities as described above (Figure **2B, C**). To automatically image zebrafish kidneys, larvae were manually positioned and oriented in the agarose cavities and subsequently imaged on a standard widefield HCS microscope. To ensure capture of entire organs and compensate for minor variations in z-positioning, each larva was acquired using z-stacks with 33 slices in the bright field and GFP channel (Figure **2D**). The cavities in the plate allow larvae to be positioned with high enough accuracy, so that regions of interest (e.g. pronephros) are located within the limited field of view for all plated embryos. Thus, the tool permits the organ and tissue specific screening on standard screening microscopes using a fixed field of view for all wells, without the necessity of additional software modules for automatic detection and centering the region of interests [16]. 

The screening system used in this study stores data as single tiff files in one folder per experimental plate. To reduce file number and thus facilitate subsequent data handling and analysis tasks, multilayer tiff files were generated for each imaging position and channel. Spatial widefield data usually suffers from out-of-focus blur thus reducing overall image quality [30]. To restore images, fluorescent datasets were batch deconvolved with Huygens Professional software using a theoretical point spread function [16]. Subsequently, maximum projection images were generated from deconvolved z-stacks (Figure **2E**). The positions of larval kidneys were automatically detected within maximum projections of deconvolved data using the center of mass of the corresponding binary image after automatic thresholding. To restrict image data to the pronephric region and remove unnecessary areas, a bounding box was defined around the center of mass and images were cropped accordingly (Figure **2F**). Subsequently, overview images were generated from cropped kidney images representing all kidneys in one 96-well plate, allowing for rapid manual assessment of morphological phenotypes (Figure **2G**). In summary, this pipeline allows consistent imaging and rapid evaluation of gross morphological abnormalities of the developing zebrafish kidney after compound treatment or in genetic screens. It can also be easily adapted for the analysis of other tissues and organs that require consistent imaging. 

### A pilot screen for drug-related effects on kidney development

The pipeline was subsequently evaluated in a pilot screen to investigate the impact of potentially nephrotoxic drugs on the development of the zebrafish pronephros. To this end, a subset of drugs from different classes was chosen for which an adverse effect on the developing kidney was previously identified in animal studies and/or human observations [2]. Dechorionated 24 hpf old embryos were treated with 8 different drugs in increasing concentrations for 24 hours. Following drug treatment, live larvae were imaged and data was visualized as described above.

To score lethality rates and development of pericardial and yolk sac edema, treated larvae were examined on a stereo microscope. The detailed results are listed in Table **S1**.

**Figure 3:**
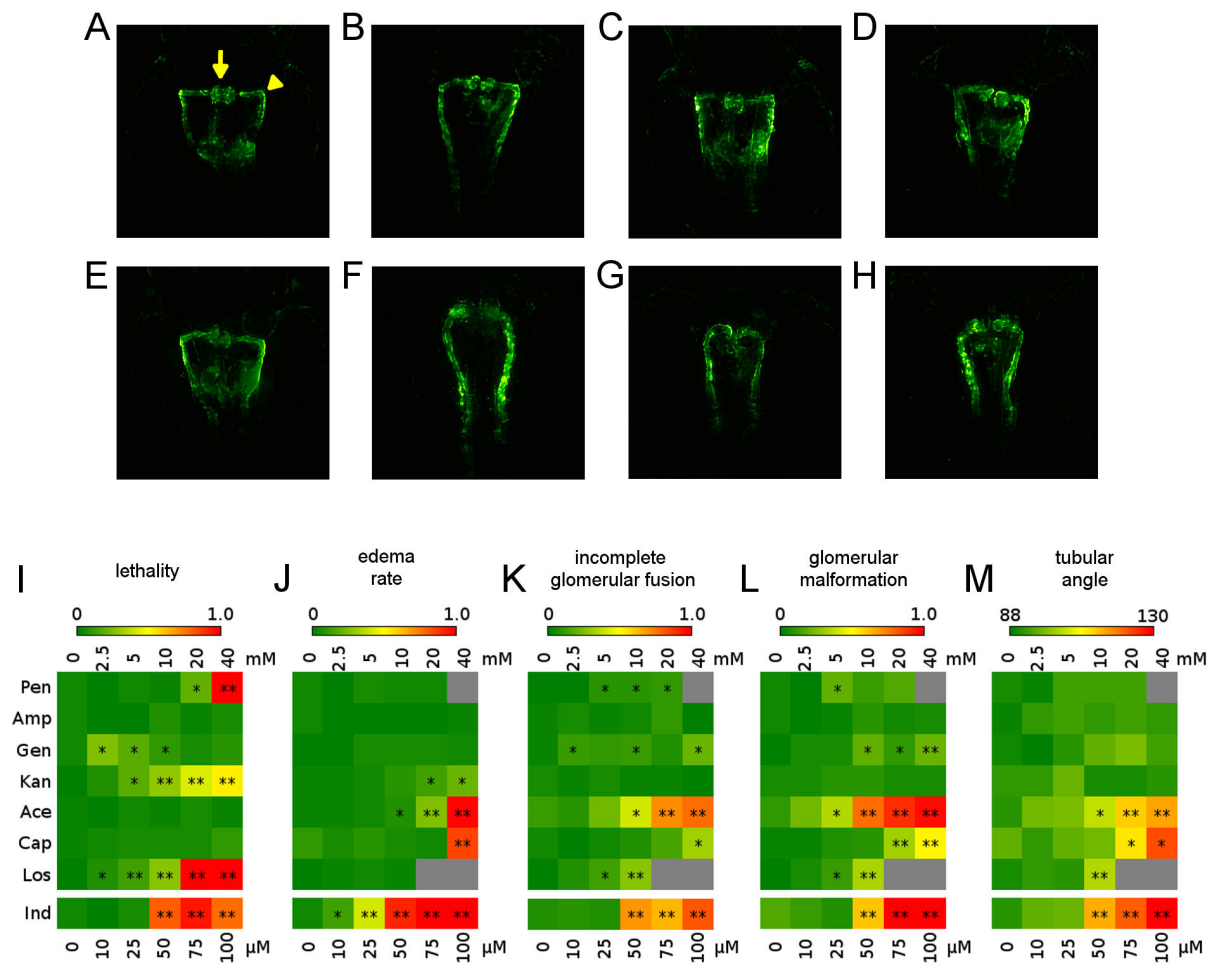
Overview of compound concentration-dependent pronephric phenotypes. Illustrative examples of pronephroi of a (A) non-treated embryo, and after treatment with (B) 20 mM penicillin, (C) 40 mM ampicillin, (D) 40 mM gentamicin, (E) 40 mM kanamycin, (F) 40 mM acetaminophen, (G) 40 mM captopril, (H) 10 mM losartan. For examples of phenotypes after Indomethacin treatment see Figure **4A-E**. Arrow and arrowheads in (A) indicate the different morphological parameters of the pronephros scored to evaluate compound effect on the developing kidney. Arrow: fused glomeruli; Arrowhead: angle between the neck segment and the proximal convoluted tubule. (I-M) Heatmaps showing (I) lethality rates, (J) edema rates and (K-M) changes in morphological parameters of the pronephros. In detail, (K) incomplete glomerular fusion, (L) glomerular malformation and (M) tubular angle. For further details see Materials and Methods and Tables **S1**-**S3**. Colour codes indicate the percentage of embryos (I-L) with particular phenotype, or the angle between neck segment and proximal convoluted tubule (M) as indicated by the colour coded legend. Grey squares indicate missing data points. Concentration ranges used are indicated above the heatmaps, or below for Indomethacin, respectively. Abbreviations: penicillin (Pen), ampicillin (Amp), gentamicin (Gen), kanamycin (Kan), acetaminophen (Ace), captopril (Cap), losartan (Los) and indomethacin (Ind). *p<0.05, **p<0.001.

To objectively quantify morphological abnormalities of the pronephros following drug treatment, glomerular and tubular alterations were discriminated in the *Tg*(*wt1b:EGFP*) transgenic line. Glomerular alterations were subdivided into (i.) glomerular malformation indicated by abnormal or reduced glomerular shape and area, and (ii.) incomplete glomerular fusion representing aberrant pronephros development. Tubular parameters were classified into (i.) the angle between the tubular neck segment and the proximal convoluted tubule and (ii.) variations in the maximum distance between the 2 tubular systems (Figure **3A**). Several drugs showed concentration dependent effects on overall survival rates, edema formation and pronephric phenotypes. Detailed results are listed in Table **S1**-**S3**. Color coded overview maps were also generated (Figure **3I-M**). To validate phenotypic alterations observed in the transgenic model and thus confirm the utility of the proposed screening pipeline, histological analysis of glomerular and tubular cross-sections of 48 hpf larvae treated at the highest non-lethal concentration was carried out (Figure **S1**).

### Impact of tested compounds on kidney development

In humans, due to the putative absence of fetal toxicity at therapeutic doses, penicillin antibiotics are widely prescribed to pregnant women and frequently administered to preterm newborns [31]. In our study, penicillin G potassium salt administration increased lethality rates dose-dependently (Figure **3I**). Concomitantly, minor pronephric alterations were observed including incompletely fused glomeruli (Figure **3B**). On the other hand, ampicillin sodium salt did not cause higher lethality, increased edema rates or major phenotypic renal alterations (Figure **3C, I-M**). Cross-sections of larvae treated with penicillin G or ampicillin did not show major glomerular or tubular alterations when compared to untreated control larvae (Figure **S1A-C**), thus confirming results obtained by fluorescence microscopy. Taken together, this indicates only minor effects of the 2 β-lactam antibiotics on pronephros development.

Aminoglycosides, although not recommended during pregnancy [31], are often used for treatment of neonatal sepsis, even in premature newborns with on-going nephrogenesis. However, serum drug concentrations can be monitored to minimize the risk of renal and auditory toxicity [32]. In our study, gentamicin sulfate administration caused only a minor increase in lethality rates without significant effects on edema formation (Figure **3I, J**). Nevertheless, glomerular malformation and incomplete glomerular fusion were found for higher drug concentrations. Tubular angle was slightly widened at higher doses (Figure **3D, M**). Histological analysis of gentamicin-treated larvae revealed no gross morphological abnormalities, although a mild rarefication of capillary loops could be seen (Figure **S1D**). Kanamycin caused a concentration-dependent increase in lethality and edema formation (Figure **3I, J**). However, glomerular and tubular parameters remained unaltered (Figure **3E**). Concordantly, no major glomerular or tubular alterations were observed in histological sections of larvae following kanamycin administration (Figure **S1E**). In other studies, microinjection of gentamicin into the cardiac venous sinus led to acute renal failure [33]. As only minor effects of gentamicin were observed in our study, it suggests that this may have been due to poorer penetration into inner organs. Several human and animal studies report on aminoglycoside-induced glomerular and tubular damage in pre- and at-term newborns [34 35–36]. Substance-specific differences in the degree of ototoxic and nephrotoxic side effects among various aminoglycosides are well known [37].

In humans, the intake of acetaminophen at therapeutic doses during gestation and administration to preterm newborns has been considered safe [38]. Its hepatotoxicity at high doses is well described [39] and has recently been investigated in zebrafish [40]. In addition, animal data further revealed fetal kidney damage following acetaminophen administration to pregnant rats [41]. In our study, acetaminophen caused concentration dependent significant alterations of pronephros morphology and an increase in edema formation, whereas lethality rates remained unchanged (Figure **3F, I-M**). Histological sections confirmed severe renal phenotypes following acetaminophen administration. No fused glomerulus was detectable ventrally to the dorsal aorta and glomerular structures appeared strongly malformed. In addition, tubular epithelium was flattened (Figure **S1F**). These results match previously published data showing dose-, duration- and onset-dependent changes in pronephros morphology following acetaminophen administration in zebrafish larvae [42].

Intake of ACE inhibitors and angiotensin receptor blockers during pregnancy has been associated with fetopathies including renal pathologies in humans [43 44–45] and other mammals [46]. In our study, captopril at 40 mM significantly increased edema formation and induced concentration dependent alterations in glomerular and tubular parameters (Figure **3G, I-M**). Losartan increased lethality rates and glomerular and tubular parameters at higher concentrations, while edema rates were unchanged (Figure **3H, I-M**). Additionally, captopril and losartan treated larvae displayed slightly altered glomerular structure in histological sections that appeared less dense compared to controls (Figure **S1G, H**).This data is partially consistent with animal studies showing renal abnormalities after treatment with ACE inhibitors or angiotensin receptors [47 48–49]. 

**Figure 4:**
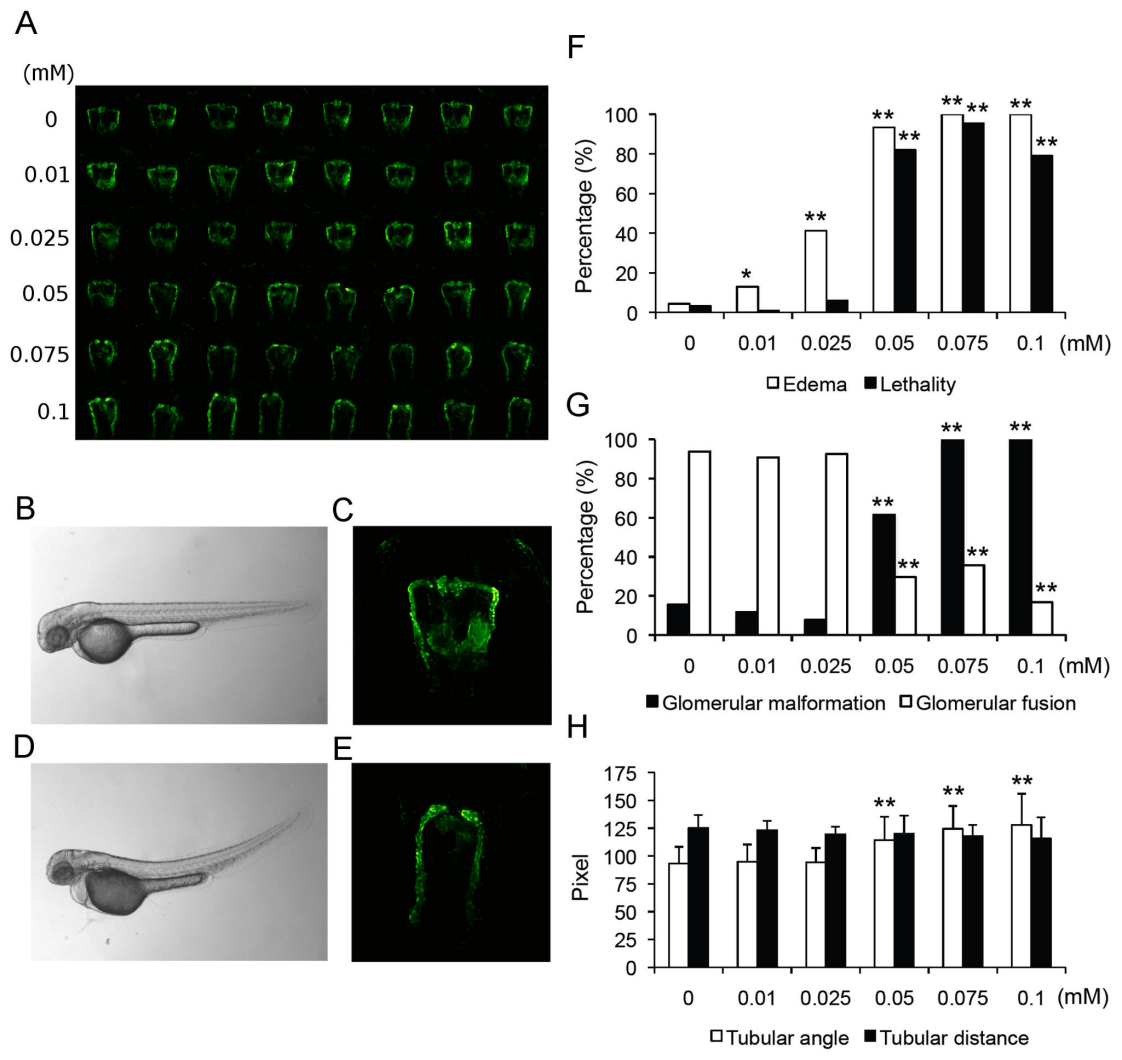
Impairment of pronephros development upon indomethacin treatment. (A) Overview of pronephric alterations in zebrafish larvae (50 hpf) following indomethacin administration for 24 hours. Row 1 shows control embryos, rows 2-6 zebrafish embryos following indomethacin administration in increasing concentrations (row 2, 0.01 mM; row 3, 0.025 mM; row 4, 0.05 mM; row 5, 0.075 mM and row 6, 0.1 mM). (B-E) Comparison of (B-C) 50 hpf control larva and (D-E) indomethacin (0.1 mM) treated larva. (D) Brightfield image shows edema formation following indomethacin administration. (E) Fluorescence image showing nephron (glomerular and tubular) malformation. (F) Quantification of lethality rates and edema formation following indomethacin administration. (G) Concentration-dependent increases in glomerular malformation and decreases in glomerular fusion rates following indomethacin administration. (H) Widened tubular angles between neck segment and proximal convoluted tubule following indomethacin administration. Data are shown as mean ± SD. *p<0.05, **p<0.001.

NSAIDs are widely used for closure of patent ductus arteriosus in preterms and are administered during pregnancy for prevention and treatment of toxemia, polyhydramnions and premature birth. However, exposure to NSAIDs during pregnancy can cause hypoperfusion of the fetal kidneys and acute renal failure in newborns with cystic changes of developing nephrons and long-term renal dysfunction [2,50]. In our study, concentrations of the non-selective COX1/COX2 inhibitor indomethacin had to be lowered due to 100% lethality at higher doses. Strikingly, even at drastically lower concentrations there was a severe phenotype (Figure **4A-E**). This included concentration dependent increases in edema formation and lethality (Figure **4D, F**). Fluorescence microscopy (Figure **4A, E**) revealed significant concentration dependent increases in glomerular malformation and incomplete glomerular fusion (Figure **4G**). Furthermore, tubular angles widened and tubular distances slightly shortened (Figure **4H**). Severe renal phenotypes were also confirmed in histological sections of indomethacin treated larvae. Here, no regular glomerulus was detectable ventrally to the dorsal aorta and laterally seen glomerular structures appeared strongly malformed or were not identifiable. Additionally, pronephric tubular epithelium appeared flattened (Figure **S1I**). Thus, our data in larval zebrafish matches previously published data from other animal models confirming severe renal side effects of indomethacin on kidney development during nephrogenesis [50].

### Automated microscopy screening of genetic kidney disease models

Beyond performing toxicological screens for kidney damage, the presented automated microscopy pipeline can be utilized in the analysis of genetic disease models. Gene-knockdown or knock-out models can potentially be used for HCS investigations for the search of therapeutic strategies for hereditary kidney diseases. To test the utility of the developed pipeline, we focused on cilia-associated human genetic disorders. 

Intraflagellar transport (IFT) constitutes the bidirectional transport of protein complexes along axonemal microtubules. IFT plays an essential role in the assembly and function of cilia and flagella by contributing to cell motility, sensory perception and cilium-based signaling [51,52]. IFT80 and IFT172 both are members of the IFT-B subcomplex [53] and while *IFT80* mutations in humans have been identified to cause Jeune asphyxiating thoracic dystrophy [23], a congenital ciliary chondrodysplasia condition associated with renal disease in approximately 20% of cases [54], no human mutations in *IFT172* have been identified to date. However, abrogation of *Ift172* function in mice leads to a VACTERL-like phenotype including renal malformations [55], indicating that IFT172 plays an important role for kidney development in mammals. In zebrafish, the insertional mutant line *ift172* ^*hi2211Tg*^ exhibits glomerular cysts and a ventral body curvature [56]. 

**Figure 5:**
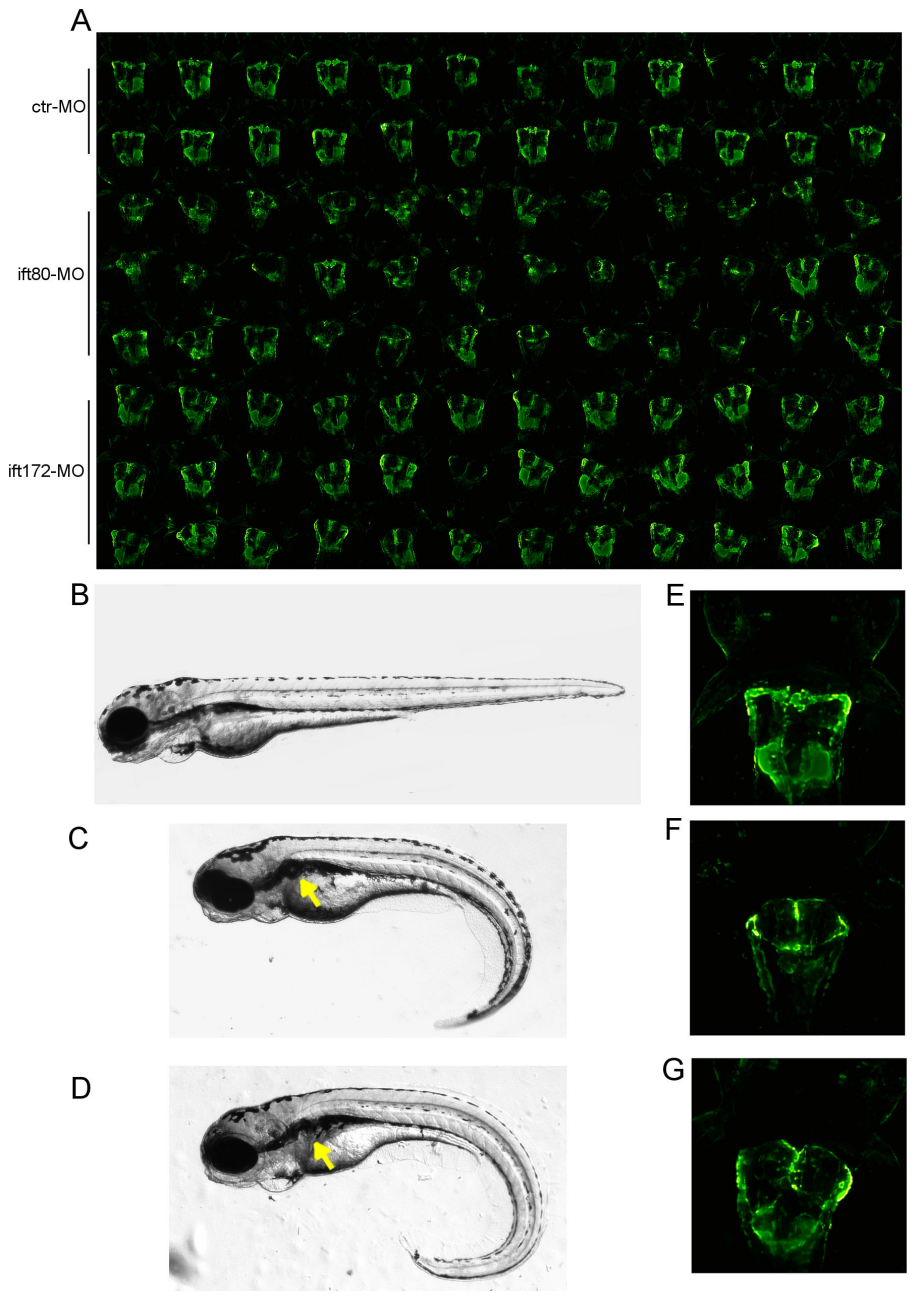
Detection of cystic kidneys after Ift80 and Ift172 knockdown. (A) Automatically generated overview thumbnail image of 96 kidneys of morpholino injected larvae: standard ctr-MO (row 1-2), ift80-MO (row 3-5) and ift172-MO (row 6-8). (B-D) Phenotypic alterations of zebrafish larvae at 4 dpf after morpholino microinjection; (C) ift80-MO and (D) ift172-MO injected embryos exhibit glomerular cyst (arrow) and a ventrally curved tail compared to (B) standard ctr-MO injected embryos. (E-G) In-detail visualisation of glomerular cysts at 72 hpf in (F) ift80-MO and (G) ift172-MO injected embryos compared to standard ctr-MO injected embryos (E) using the Tg(wt1b:EGFP) transgenic line.

Splice blocking morpholinos for *ift80* and *ift172* were designed as described in the Methods section. By using the standard positioning tool as described above, automated imaging of dorsally positioned morpholino-injected *Tg*(*wt1b:EGFP*) zebrafish (3 dpf) was performed in 96 well plates (Figure **5A**). Microscopy revealed a ventral curvature of the tail (Figure **5B-D**) affecting approximately 90% of all morpholino injected embryos and consistent with the phenotype previously described for *ift80* morphants [23]. Morphological alterations in fluorescence microscopy predominantly consisted of large cystic glomeruli (Figure **5E-G**) that were consistently reproducible [23], while embryos treated with standard morpholino showed normal glomerular morphology. Cross-sections of both ift80- and ift172-morpholino injected *Tg*(*wt1b:EGFP*) zebrafish confirmed the formation of large pronephric cysts (Figure **S2A-C**). Tubular dilatation and epithelial flattening was observed both in fluorescence images (Figure **5F, G**) and histological sections (Figure **S2B, C**). This further demonstrates that our pipeline is suitable for large scale therapeutic screening investigations in genetic models of renal disease such as ciliopathies. However, the applicability largely depends on the morphological phenotypes as severe malformations impair position accuracy within cavities.

## Conclusions

Here, we demonstrate the development of an automated screening pipeline for imaging developing kidneys in the zebrafish larvae. This novel methodology allows for the consistent acquisition of dorsal views of pronephric kidneys on a standard inverted screening microscope. The platform can serve as a convenient tool in kidney research, e.g. in chemical studies as a primary screening tool to identify organotoxic substances or to search for potential therapeutic compounds that attenuate renal pathology in disease models. Importantly, the imaging protocol is easy-to-use and can be readily modified for studying other organ systems or tissues such as the brain region. Furthermore, in combination with HCS software tools which enable automated feature of interest detection, it can be used for the automated acquisition of standardized multidimensional high resolution datasets.

The pilot screen for nephrotoxic drugs during nephrogenesis revealed a concentration dependent effect of several compounds on nephrogenesis. Thus, considering the lingering lack of data for many substances, subsequent large-scale investigations performed with this screening pipeline might contribute to our understanding of substance-specific nephrotoxic side effects. 

The current pipeline only allows for scoring of gross morphological abnormalities of the pronephros. Therefore, we validated the impact of treatments on pronephros formation by histological analysis, which largely confirmed the phenotypes observed in the transgenic model. This further demonstrates the utility of the established screening pipeline to score pronephric phenotypes. Moreover, the histological analysis revealed additional alterations, such as epithelial flattening, which are more difficult to score in the fluorescence data, thus complementing the *in vivo* approach. Although protocols for large scale histology experiments exist [57], the fast filtering of compound libraries by *in vivo* screening is considerably more time- and cost effective. Therefore, we propose that in a genuine large scale experiment, histological analysis is only carried out as a follow-up experiment in combination with molecular methods to characterize hits identified in a screen. 

The mode of drug administration imposes another limitation of the established pipeline, as penetration to inner organs can be hampered by the chemical and physical properties of the noxa and the biological barrier of the larval zebrafish skin. This can be overcome by microinjection of drugs into the blood stream as described by Hentschel et al. [33]. Duration of drug treatment and its transferability to the human situation is another limitation of the pilot screen in zebrafish. In zebrafish, glomerular filtration starts around 48 hpf and the pronephros is fully matured at 4 dpf [58]. Hence, due to the rapid embryogenesis of the zebrafish, future studies have to employ different treatment periods to analyze the impact of compounds at the different stages of nephrogenesis [42]. 

Genetic research over the last years has demonstrated that the zebrafish pronephros is a valuable model system for the study of hereditary human nephropathies as abnormalities in podocyte gene function, renal epithelial primary cilia genes and renal ion channels and transporters lead to defective pronephric kidney function in the zebrafish mimicking human disease [59]. However, screening for disease modulating compounds in a zebrafish model requires convenient and accessible protocols. Here, we demonstrate that the developed imaging pipeline can also be utilized to detect abnormal phenotypes in genetic disease models. Thus, it could serve as a platform for prospective high-content drug screening experiments.

Finally, for genuine high content screening, an automated image analysis pipeline for extracted morphological features would be highly beneficial [22,60]. Moreover, compounds influencing kidney function without altering pronephros morphology cannot be identified using this pipeline. Thus, protocols for the large scale analysis of kidney function need to be developed or modified to be compatible with automated imaging assays, respectively [61]. 

## Supporting Information

Figure S1 **Cross-sections of pronephric regions after compound exposure.** Shown are glomerular (upper panels) and tubular (lower panels) sections at 48 hours post fertilization. (A) control, (B) penicillin (20 mM), (C) ampicillin (40 mM), (D) gentamicin (40 mM), (E) kanamycin (40 mM), (F) acetaminophen (40 mM), (G) captopril (40 mM), (H) losartan (10 mM) and (I) indomethacin (0.75 mM) treatment.
(TIF)

Figure S2 **Cross-sections of pronephric regions after morpholino injections.** Shown are glomerular (upper panels) and tubular (lower panels) sections at 72 hours post fertilization. (A) control-MO, (B) ift80-MO and (C) ift172-MO injection.
(TIF)

Table S1 **Lethality and edema formation in zebrafish larvae following drug treatment.**
(DOCX)

Table S2 **Glomerular alterations in zebrafish larvae following drug treatment.**
(DOCX)

Table S3 **Tubular angle and distance in zebrafish larvae following drug treatment.**
(DOCX)

Macro S1 **Fiji macro to crop images and generate overview images.**
(ZIP)
