# Supplementary figures and images for: Development of an Automated Imaging Pipeline for the Analysis of the Zebrafish Larval Kidney
Source: PLoS One. 2013 Dec 4;8(12):e82137. doi: 10.1371/journal.pone.0082137 (PMC3852951; doi:10.1371/journal.pone.0082137)

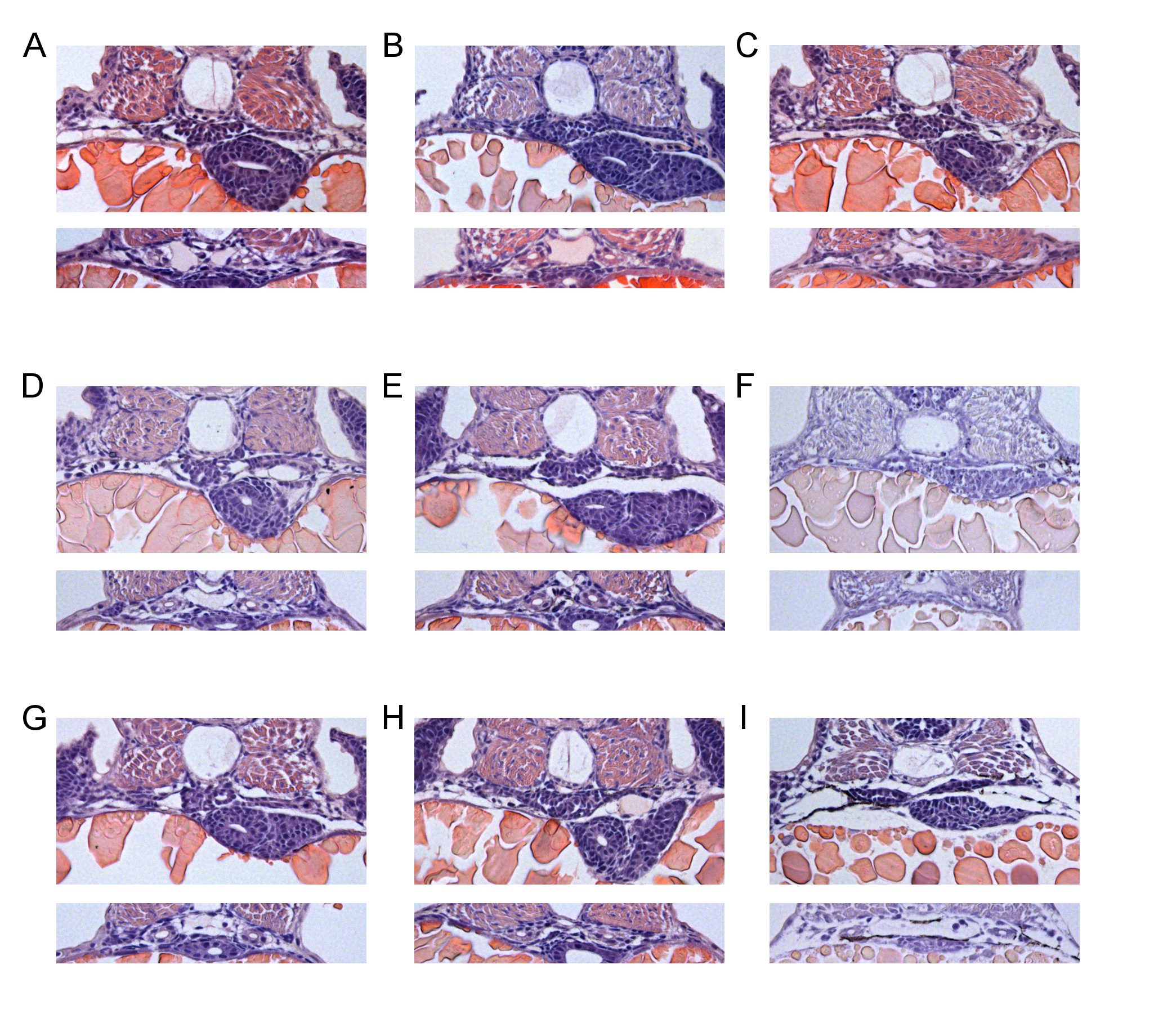

Supplement: Figure S1 — Cross-sections of pronephric regions after compound exposure. Shown are glomerular (upper panels) and tubular (lower panels) sections at 48 hours post fertilization. (A) control, (B) penicillin (20 mM), (C) ampicillin (40 mM), (D) gentamicin (40 mM), (E) kanamycin (40 mM), (F) acetaminophen (40 mM), (G) captopril (40 mM), (H) losartan (10 mM) and (I) indomethacin (0.75 mM) treatment. (TIF) [file pone.0082137.s001.tif]

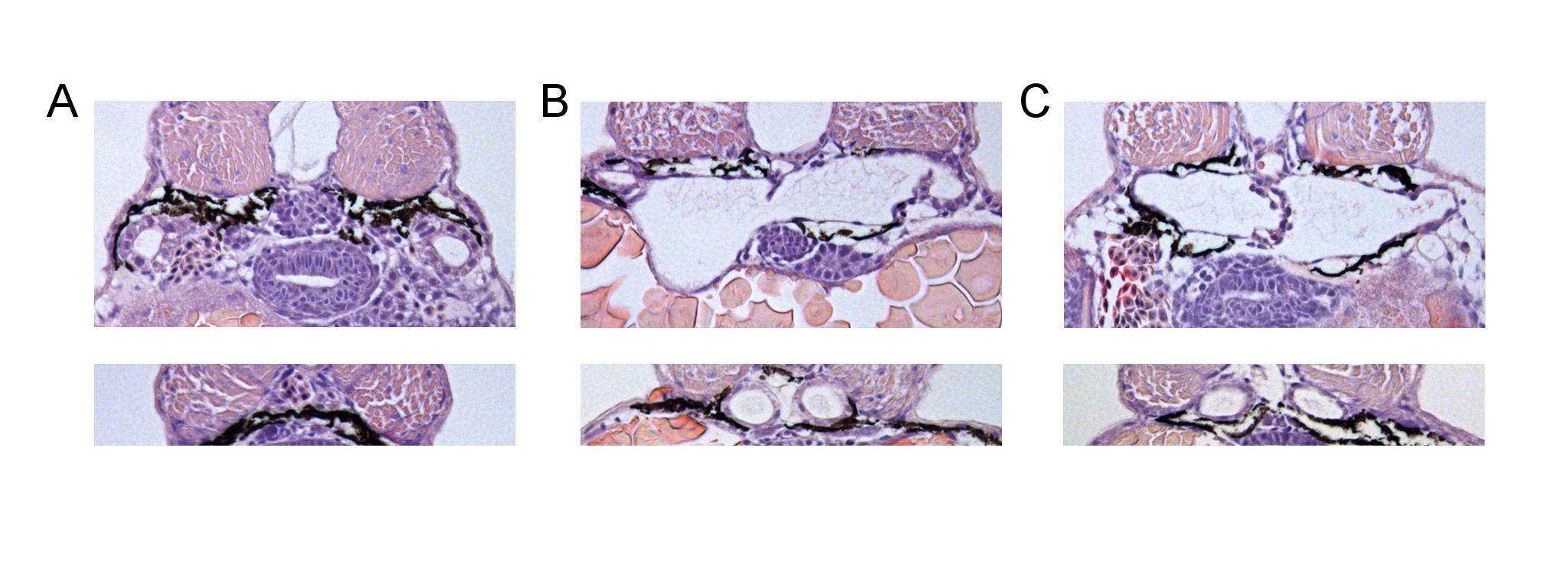

Supplement: Figure S2 — Cross-sections of pronephric regions after morpholino injections. Shown are glomerular (upper panels) and tubular (lower panels) sections at 72 hours post fertilization. (A) control-MO, (B) ift80-MO and (C) ift172-MO injection. (TIF) [file pone.0082137.s002.tif]
