# Supplementary material for: Development of an Automated Imaging Pipeline for the Analysis of the Zebrafish Larval Kidney
Source: PLoS One. 2013 Dec 4;8(12):e82137. doi: 10.1371/journal.pone.0082137 (PMC3852951; doi:10.1371/journal.pone.0082137)
Supplement: Table S1 — Lethality and edema formation in zebrafish larvae following drug treatment. (DOCX) [file pone.0082137.s003.docx]

**Table S1. Lethality and edema formation in zebrafish larvae following drug treatment.**

Table S1a.

|  | **Lethality (%)** | | | | | | **Edema (%)** | | | | | |
| --- | --- | --- | --- | --- | --- | --- | --- | --- | --- | --- | --- | --- |
| **Concentration (mM)** | **0** | **2.5** | **5** | **10** | **20** | **40** | **0** | **2.5** | **5** | **10** | **20** | **40** |
| Penicillin | 4.2  (n=95) | 0  (n=76) | 4.6  (n=152) | 2.0  (n=153) | 19.8*  (n=101) | 100**  (n=83) | 4.4  (n=91) | 1.3  (n=76) | 4.8  (n=145) | 4.7  (n=150) | 3.7  (n=81) | N/A |
| Ampicillin | 3.9  (n=51) | 0  (n=66) | 0  (n=72) | 6.8  (n=74) | 2.9  (n=68) | 5.3  (n=57) | 4.1  (n=49) | 1.5  (n=66) | 1.4  (n=72) | 1.5  (n=69) | 1.5  (n=66) | 1.9  (n=54) |
| Gentamicin | 4.0  (n=149) | 26.3*  (n=19) | 17.2*  (n=87) | 10.4*  (n=164) | 5.5  (n=163) | 9.2  (n=153) | 1.4  (n=143) | 0  (n=14) | 5.6  (n=72) | 5.4  (n=147) | 5.2  (n=154) | 4.1  (n=147) |
| Kanamycin | 1.5  (n=66) | 6.8  (n=88) | 18.6* (n=70) | 29.0** (n=76) | 42.2** (n=90) | 53.2** (n=79) | 3.1  (n=65) | 2.4  (n=82) | 3,5  (n=57) | 9,3  (n=54) | 13,5* (n=52) | 21,6* (n=37) |
| Acetaminophen | 1.9 (n=156) | 0.8 (n=133) | 3.2 (n=126) | 1.6 (n=124) | 5.4 (n=130) | 2.2 (n=137) | 2.0 (n=153) | 2.3 (n=132) | 4.1 (n=122) | 7.4* (n=122) | 26.0** (n=123) | 98.5** (n=134) |
| Captopril | 1.8  (n=56) | 3.7  (n=54) | 5.0  (n=60) | 5.2  (n=77) | 5.6  (n=72) | 9.9  (n=81) | 10.9 (n=55) | 5.8  (n=52) | 8.8  (n=57) | 5.5  (n=73) | 4.4  (n=68) | 86.3** (n=73) |
| Losartan | 0  (n=132) | 6.8* (n=132) | 13.1** (n=153) | 27.8** (n=180) | 100** (n=135) | 100** (n=110) | 1.5 (n=132) | 1.6 (n=123) | 4.5 (n=133) | 2.3 (n=130) | N/A | N/A |

*p<0.05 vs. 0 mM (control), **p<0.001 vs. 0 mM (control), N/A: not available.

Table S1b.

|  | **Lethality (%)** | | | | | | **Edema (%)** | | | | | |
| --- | --- | --- | --- | --- | --- | --- | --- | --- | --- | --- | --- | --- |
| **Concentration (mM)** | **0** | **0.01** | **0.025** | **0.05** | **0.075** | **0.1** | **0** | **0.01** | **0.025** | **0.05** | **0.075** | **0.1** |
| Indomethacin | 3.2  (n=95) | 0.9 (n=117) | 6.0 (n=116) | 81.9** (n=83) | 95.5** (n=88) | 79.1** (n=129) | 4.4  (n=92) | 12.9* (n=116) | 41.3** (n=109) | 93.3** (n=15) | 100** (n=4) | 100** (n=27) |

*p<0.05 vs. 0 mM (control), **p<0.001 vs. 0 mM (control).
