# Supplementary material for: Development of an Automated Imaging Pipeline for the Analysis of the Zebrafish Larval Kidney
Source: PLoS One. 2013 Dec 4;8(12):e82137. doi: 10.1371/journal.pone.0082137 (PMC3852951; doi:10.1371/journal.pone.0082137)
Supplement: Table S2 — Glomerular alterations in zebrafish larvae following drug treatment. (DOCX) [file pone.0082137.s004.docx]

**Table S2. Glomerular alterations in zebrafish larvae following drug treatment.**

Table S2a.

|  | **Glomerular malformation (%)** | | | | | | **Incomplete glomerular fusion (%)** | | | | | |
| --- | --- | --- | --- | --- | --- | --- | --- | --- | --- | --- | --- | --- |
| **Concentration (mM)** | **0** | **2,5** | **5** | **10** | **20** | **40** | **0** | **2,5** | **5** | **10** | **20** | **40** |
| Penicillin | 4.4 (n=46) | 0  (n=46) | 18.8* (n=48) | 11.9 (n=42) | 16.7 (n=48) | N/A | 0  (n=47) | 0  (n=46) | 8.2* (n=49) | 9.3* (n=43) | 10.2* (n=49) | N/A |
| Ampicillin | 4.6 (n=44) | 0  (n=36) | 2.7 (n=37) | 9.3 (n=43) | 4.6 (n=44) | 5.1 (n=39) | 2.3 (n=44) | 5.3 (n=38) | 2.6 (n=38) | 4.4 (n=45) | 10.9 (n=46) | 0  (n=39) |
| Gentamicin | 0 (n=54) | 5.7 (n=53) | 5.2 (n=58) | 19.3* (n=57) | 10.9* (n=55) | 21.3** (n=47) | 1.8 (n=56) | 11.3* (n=53) | 10.2 (n=59) | 11.7* (n=60) | 7.1 (n=56) | 20.4* (n=49) |
| Kanamycin | 5.3 (n=38) | 5.3 (n=38) | 8.1 (n=37) | 7.9 (n=38) | 5.7 (n=35) | 5.9 (n=34) | 5.3 (n=38) | 2.6 (n=38) | 5.3 (n=38) | 2.6 (n=39) | 2.9 (n=34) | 0  (n=36) |
| Acetaminophen | 9.8 (n=41) | 22.9 (n=35) | 35.9* (n=39) | 78.4** (n=37) | 91.7** (n=24) | 96.9** (n=32) | 11.9 (n=42) | 7.9 (n=38) | 22.2 (n=36) | 40.6* (n=32) | 73.1** (n=26) | 78.8** (n=33) |
| Captopril | 2.2 (n=46) | 2.3 (n=44) | 6.5 (n=46) | 8.7 (n=46) | 31.8** (n=44) | 52.5** (n=40) | 4.3 (n=47) | 0  (n=44) | 4.7 (n=43) | 6.5 (n=46) | 15.6 (n=45) | 31.7* (n=41) |
| Losartan | 1.5 (n=65) | 5.0 (n=60) | 11.9* (n=67) | 34.2** (n=41) | N/A | N/A | 1.5 (n=65) | 6.8 (n=59) | 13.4* (n=67) | 27.9** (n=43) | N/A | N/A |

*p<0.05 vs. 0 mM (control), **p<0.001 vs. 0 mM (control), N/A: not available.

Table S2b.

|  | **Glomerular malformation (%)** | | | | | | **Incomplete glomerular fusion (%)** | | | | | |
| --- | --- | --- | --- | --- | --- | --- | --- | --- | --- | --- | --- | --- |
| **Concentration (mM)** | **0** | **0.01** | **0.025** | **0.05** | **0.075** | **0.1** | **0** | **0.01** | **0.025** | **0.05** | **0.075** | **0.1** |
| Indomethacin | 15.6 (n=32) | 11.8 (n=34) | 7.7 (n=39) | 61.5** (n=26) | 100** (n=14) | 100** (n=6) | 6.3 (n=32) | 9.1 (n=33) | 7.5 (n=40) | 70.4** (n=27) | 64.3** (n=14) | 83.3** (n=6) |

*p<0.05 vs. 0 mM (control), **p<0.001 vs. 0 mM (control).
