# Supplementary material for: Development of an Automated Imaging Pipeline for the Analysis of the Zebrafish Larval Kidney
Source: PLoS One. 2013 Dec 4;8(12):e82137. doi: 10.1371/journal.pone.0082137 (PMC3852951; doi:10.1371/journal.pone.0082137)
Supplement: Table S3 — Tubular angle and distance in zebrafish larvae following drug treatment. (DOCX) [file pone.0082137.s005.docx]

**Table S3. Tubular angle and distance in zebrafish larvae following drug treatment.**

Table S3a.

|  | **Tubular Angle (°)** | | | | | | **Tubular Distance (a.u.)** | | | | | |
| --- | --- | --- | --- | --- | --- | --- | --- | --- | --- | --- | --- | --- |
| **Concentration (mM)** | **0** | **2.5** | **5** | **10** | **20** | **40** | **0** | **2.5** | **5** | **10** | **20** | **40** |
| Penicillin | 90.2  +/- 13.0 (n=47) | 88.6  +/- 11.4 (n=49) | 93.3  +/- 16.5 (n=50) | 93.1  +/- 14.5 (n=47) | 93.1  +/- 14.1 (n=47) | N/A | 121.3  +/- 14.2 | 123.4  +/- 8.9 | 118.1  +/- 15.4 | 118.9  +/- 15.3 | 116.8  +/- 8.6 | N/A |
| Ampicillin | 88.4  +/- 11.5 (n=34) | 92.0  +/- 12.9 (n=36) | 92.7  +/- 12.7 (n=36) | 92.1  +/- 17.9 (n=42) | 92.9  +/- 15.5 (n=39) | 91.8  +/- 14.2 (n=40) | 125.1  +/- 8.6 | 119.9  +/- 14.0 | 118.1  +/- 15.2* | 115.1  +/- 13.9 | 115.9  +/- 13.6 | 118.3  +/- 13.3 |
| Gentamicin | 89.4  +/- 11.3 (n=55) | 87.7  +/- 13.1 (n=11) | 91.3  +/- 12.7 (n=38) | 95.3  +/- 18.5 (n=57) | 97.2  +/- 17.3* (n=58) | 92.8  +/- 11.9 (n=60) | 138.0  +/- 30.0 | 128.6  +/- 8.3 | 138.0  +/- 33.0 | 129.2  +/- 24.7 | 129.6  +/- 27.0 | 130.6  +/- 26.8 |
| Kanamycin | 91.7  +/- 11.4 (n=38) | 92.1  +/- 13.9 (n=41) | 96.4  +/- 19.2 (n=41) | 90.2  +/- 12.1 (n=39) | 89.8  +/- 10.9 (n=34) | 90.9  +/- 15.8 (n=37) | 120.5  +/- 8.3 | 116.6  +/- 12.5 | 111.5  +/- 14.1* | 117.8  +/- 10.6 | 118.2  +/- 11.8 | 116.2  +/- 14.7 |
| Acetaminophen | 90.9  +/- 12.3 (n=42) | 97.0  +/- 15.1 (n=39) | 97.5  +/- 14.7 (n=40) | 103.1  +/- 23.0* (n=37) | 111.9  +/- 18.6** (n=27) | 114.7  +/- 24.0** (n=32) | 140.2  +/- 29.9 | 138.3  +/- 28.8 | 135.4  +/- 26.7 | 134.3  +/- 31.1 | 130.4  +/- 27.4 | 128.6  +/- 31.0 |
| Captopril | 95.5  +/- 15.1 (n=31) | 91.5  +/-11.0 (n=30) | 95.3  +/- 16.5 (n=31) | 96.8  +/- 11.6 (n=30) | 110.0  +/- 19.3* (n=31) | 121.6  +/- 25.2* (n=27) | 150.2  +/- 33.7 | 147.6  +/- 34.6 | 147.5  +/- 37.1 | 146.0  -/- 32.8 | 141.8  +/- 26.0 | 133.0  +/- 26.0 |
| Losartan | 89.6  +/- 12.8 (n=77) | 91.7  +/- 14.9 (n=66) | 91.4  +/- 16.1 (n=66) | 102.0  +/- 22.7** (n=42) | N/A | N/A | 118.0  +/- 10.8 | 118.2  +/- 11.7 | 115.6  +/- 12.3 | 103.5  +/- 13.4** | N/A | N/A |

*p<0.05 vs. 0 mM (control), **p<0.001 vs. 0 mM (control). a.u.: arbitrary units. N/A: not available. Data is shown as mean +/- SD.

Table S3b.

|  | **Tubular Angle (°)** | | | | | | **Tubular Distance (a.u.)** | | | | | |
| --- | --- | --- | --- | --- | --- | --- | --- | --- | --- | --- | --- | --- |
| **Concentration (mM)** | **0** | **0.01** | **0.025** | **0.05** | **0.075** | **0.1** | **0** | **0.01** | **0.025** | **0.05** | **0.075** | **0.1** |
| Indomethacin | 92.0  +/- 13.8 (n= 53) | 95.8  +/- 15.2 (n= 90) | 96.5  +/- 12.5 (n= 62) | 114.8  +/- 20.1** (n=46) | 120.9  +/- 21.1** (n=26) | 127.7  +/- 28.2** (n=12) | 123.4  +/- 10.6 (n=29) | 121.9  +/- 8.2 (n=46) | 116.4  +/- 8.1 (n=32) | 119.5  +/- 15.8 (n=25) | 118.5  +/- 9.3 (n=14) | 115.5  +/- 19.5 (n=6) |

*p<0.05 vs. 0 mM (control), **p<0.001 vs. 0 mM (control). a.u.: arbitrary units. Data is shown as Mean +/- SD.
